# Multi‐Omics Combined With Mitochondrial Feeding Assays Reveal a Novel Energy Metabolism Strategy for Floral Thermogenesis in Magnolia Driven by Synergistic Supply of Multiple Substrates

**DOI:** 10.1111/pbi.70479

**Published:** 2025-11-28

**Authors:** Siqin Wang, Jiying Li, Zhang Wang, Miao Yu, Chang Liu, Dongye Liu, Ruohan Wang

**Affiliations:** ^1^ State Key Laboratory of Tree Genetics and Breeding, National Engineering Research Center of Tree Breeding and Ecological Restoration, Key Laboratory for Genetics and Breeding of Forest Trees and Ornamental Plants, Ministry of Education, College of Biological Sciences and Biotechnology Beijing Forestry University Beijing P. R. China

**Keywords:** energy metabolism, fatty acid β‐oxidation, mitochondrial feeding, mitochondrial pyruvate carrier, NAD‐malic enzyme

## Abstract

The high demand for energy during floral thermogenesis drives the synergistic operation of multiple energy substrates for the rapid temperature rise and successful reproduction of flowers. However, how thermogenic plants precisely regulate substrate supply and metabolic pathways within a short time to support large‐scale energy and heat release remains a mystery. This study revealed the elaborate synergistic supply mechanism of multi‐source substrates in *Magnolia denudata* during thermogenesis. Transcriptome analysis showed that genes related to the tricarboxylic acid (TCA) cycle and oxidative phosphorylation (OXPHOS) were significantly upregulated during the thermogenic stage (S2). Mitochondrial feeding assays using isotopically labelled substrates revealed that during the thermogenic stage, both the amount of pyruvate imported via the mitochondrial pyruvate carrier (MPC) and NAD‐malic enzyme (NAD‐ME) increased, and their synergistic effect accelerated the metabolic flow of the TCA cycle. Targeted lipidomics analysis indicated that the content of 63.6% fatty acids in the fatty acid degradation pathway decreased, while the key enzyme genes involved in triacylglycerol lipase (TGL) and fatty acid β‐oxidation pathways were highly expressed during the thermogenic stage. In addition, enhanced expression of genes related to alanine aminotransferase (AlaAT) and glutamate dehydrogenase (GDH) suggested that amino acid metabolism might provide additional substrates for thermogenesis. This study clarifies the synergistic energy supply of carbohydrate, fatty acid and amino acid metabolism during thermogenesis in *M. denudata* , providing new evidence for understanding the metabolic regulatory flexibility of floral thermogenesis in plants.

## 1 Introduction

Floral thermogenesis refers to the ability of some plant species to autonomously produce heat and regulate their own temperature during flowering, thereby maintaining the temperature of internal floral organs higher than the ambient temperature (Wang et al. 2013; Yu, Wang, Gu, et al. 2025). Thermogenesis in plant reproductive organs has multiple biological significances. Firstly, it promotes the release of floral volatile compounds, attracting insect pollinators to visit flowers (Wang et al. 2014, 2022). Secondly, thermogenesis provides pollinators with warm floral chambers as a reward (Seymour and Matthews 2006). Additionally, it facilitates pollen germination and pollen tube growth, ensuring successful reproduction (Bian et al. 2021). Although all plant organs generate heat during metabolism, this process is relatively slow in most plants, and the heat is quickly dissipated, making it difficult to observe a significant temperature rise (Seymour and Schultze‐Motel 1997). In contrast, the floral organs of thermogenic plants can release a large amount of heat in a short period, resulting in a local temperature significantly higher than the surrounding environment. This undoubtedly relies on complex and orderly processes of rapid substrate mobilisation and efficient energy metabolism. However, previous studies have rarely focused on how multi‐source substrates are synergistically metabolised to meet the high energy demand of floral thermogenesis.

Pyruvate, as a key product of the glycolysis pathway, plays a crucial role in cellular energy supply (Tang et al. 2022). Under the catalysis of the pyruvate dehydrogenase (PDH) complex, pyruvate is converted to acetyl‐CoA, which enters the tricarboxylic acid (TCA) cycle (Lee et al. 2020; Le et al. 2021; Tcherkez et al. 2024). As a core link in energy production through cellular respiration, the TCA cycle completely oxidises and decomposes acetyl‐CoA via a series of enzymatic reactions, accompanied by the generation of large amounts of NADH and FADH_2_ (Ducat 2015). These reducing equivalents transfer electrons in the electron transport chain, driving proton transmembrane transport to form a proton electrochemical gradient, which in turn promotes ATP synthase to synthesise a large quantity of ATP, providing sufficient energy for cells (Fernie et al. 2004). Notably, alternative oxidase (AOX) can bypass some phosphorylation sites in the electron transport chain, allowing electrons to be directly transferred to oxygen, with energy released in the form of heat (Van Aken et al. 2009). This not only effectively avoids feedback inhibition of metabolism caused by excessive ATP accumulation but also provides a stable heat source for maintaining the continuous process of plant thermogenesis.

Most of the pyruvate in plant mitochondria is derived from three main pathways: (1) it is transported from the cytoplasm to the mitochondrial matrix via the mitochondrial pyruvate carrier (MPC); (2) it is synthesised in the mitochondrial matrix by alanine aminotransferase (AlaAT); and (3) it is synthesised in the mitochondrial matrix through the oxidative decarboxylation of malate catalysed by NAD‐malic enzyme (NAD‐ME) (Le et al. 2022). These pyruvate pools can be dynamically reorganised according to the cytosolic pyruvate transport rate, exerting differential effects on the distribution of mitochondrial organic acid metabolic fluxes and energy production during plant aerobic respiration. Studies have shown that in *Arabidopsis thaliana* , the synergistic action of MPC and AlaAT provides most of the pyruvate for the TCA cycle, whereas NAD‐ME contributes minimally to pyruvate supply both in vivo and in vitro. However, mitochondria isolated from the mitochondrial pyruvate carrier 1 (MPC1) loss‐of‐function mutant (*mpc1*) exhibit a higher rate of NAD‐ME‐dependent pyruvate production (Le et al. 2021), suggesting the existence of a metabolic switching mechanism. Pyruvate generated by NAD‐ME acts as an emergency valve, which is only incorporated into the TCA cycle when the pyruvate transported into the mitochondrial matrix is insufficient to meet cellular energy demands. Given that thermogenesis consumes large amounts of energy, plants must efficiently coordinate various internal metabolic pathways to meet this demand. Therefore, the switching mechanism underlying pyruvate supply during thermogenesis in *Magnolia denudata* warrants further investigation.

In addition to pyruvate, fatty acids and amino acids are also efficient energy substrates in plants (Porras‐Dominguez et al. 2024). Fatty acids are gradually degraded via the β‐oxidation pathway to generate large amounts of acetyl‐CoA (Jallet et al. 2020), which directly enters the TCA cycle for complete oxidation to release energy. Compared with other substrates, the complete oxidation of fatty acids produces a greater amount of ATP (Moczulski et al. 2009). This pathway plays an important role in energy supply during seed germination and serves as a crucial energy source when plants respond to stress (Xiang et al. 2023; Xiao et al. 2023). Furthermore, some amino acids can be converted into intermediate products that enter the TCA cycle through reactions such as deamination, providing an additional energy source for cellular respiration. For example, glutamine (Gln) is converted to glutamate (Glu) via deamination, and the latter is further converted to 2‐oxoglutarate (2‐OG) under the catalysis of glutamate dehydrogenase (GDH) to enter the TCA cycle for energy supply (Qiu et al. 2019). This also realises the coupling of nitrogen metabolism and respiratory carbon metabolism. Although the roles of fatty acid oxidation and glutamate catabolism in plant energy supply have been extensively studied, their potential contributions to plant thermogenesis have received little attention.

This study aims to explore how thermogenic plants regulate the supply and metabolic pathways of multiple energy substrates such as pyruvate, fatty acids and amino acids to meet the high energy demand during thermogenesis. Through time‐series transcriptome analysis, the key gene regulatory patterns of the TCA cycle, oxidative phosphorylation and glycolysis pathways were systematically identified. By isolating the mitochondria of the gynoecium at the pre‐thermogenic (S1) and peak thermogenic (S2) stages and conducting labelled and unlabelled substrate feeding experiments, the changes in pyruvate flux mediated by MPC and NAD‐ME under different metabolic states were quantitatively analysed. The combination of targeted lipidomics and time‐series transcriptome analysis revealed that fatty acid β‐oxidation may be involved in substrate supply during thermogenesis. In addition, the potential role of alanine and Glu as additional energy substrates was preliminarily explored. This study presents the diversity of substrate supply and the flexibility of metabolic regulation in *M. denudata* during thermogenesis.

## 2 Materials and Methods

### 2.1 Plant Materials

Fresh flowers were collected from *Magnolia denudata* Desr. (Magnoliaceae) plants growing on the campus of Beijing Forestry University (40°00′03″ N, 116°20′25″ E, a.s.l., 68 m). *M. denudata* flowers at different developmental stages were accurately identified based on morphological characteristics and infrared thermal imaging results. Our previous study has confirmed that the gynoecium is the main thermogenic tissue (Wang et al. 2013; Wang, Yu, Wang, et al. 2025). Therefore, gynoecium samples at five complete developmental stages – S0 (petal‐unexposed stage), S1 (pre‐thermogenic stage), S2 (thermogenic peak stage), S3 (post‐thermogenic peak stage) and S4 (wilting stage) – were selected for time‐series transcriptome analysis, metabolite content determination and β‐amylase activity assay. Gynoecium samples at the pre‐thermogenic (S1) and peak thermogenic (S2) stages were used for mitochondrial feeding experiments and targeted lipidomics analysis.

### 2.2 Time‐Series Transcriptomic Analysis

Total RNA was extracted using the method described in the RNeasy Plant Mini Kit (QIAGEN). After RNA extraction and cDNA library construction, paired‐end sequencing was performed on the Illumina Novaseq 6000 platform. Raw reads were filtered using the fastp software, and clean reads were aligned to the reference genome of *Magnolia biondii* Pamp. (Dong et al. 2021) using the Hisat2 software (Kim et al. 2019). Gene expression levels were evaluated using fragments per kilobase of transcript per million mapped reads (FPKM). Differentially expressed genes (DEGs) were identified by pairwise comparisons among the five sample groups using the DESeq2 software (Love et al. 2014). Genes with |log_2_(fold change)| > 1 and FDR ≤ 0.05 were considered differentially expressed. All raw RNA‐Seq data from this study have been deposited in the NCBI Sequence Read Archive under the accession number PRJNA1182092.

### 2.3 Determination of Energy Substrate Contents and β‐Amylase Activity

The contents of starch, glucose, triacylglycerol (TG), glutamate (Glu) and β‐amylase activity were measured using kits purchased from Beijing Solarbio Science & Technology Co. Ltd. Fresh gynoecium tissues of *M. denudata* were collected and processed according to the kit instructions. Each metabolite and β‐amylase activity was quantified by measuring the absorbance at their characteristic absorption peaks.

### 2.4 Mitochondrial Isolation and Substrate Feeding

Collect 0.1 g of gynoecia from *M. denudata* flowers, chop them into pieces with a double‐edged blade and place them in a pre‐cooled mortar. Add 2 mL of mitochondrial extraction buffer (2 mM EDTA, 10 mM K_2_PO_4_, 1% BSA, 0.3 M sucrose, 25 mM Na_4_P_2_O_7_, 1% PVP‐40, 4 mM L‐cysteine, 20 mM L‐ascorbic acid, pH = 7.5) and grind to form a homogenate. The entire process is performed on ice to maintain mitochondrial activity. The homogenate is filtered through a 20 μm nylon mesh to remove disrupted cells. Then, the filtrate is centrifuged at 2000 *g* for 10 min at 4°C. The supernatant is carefully transferred and centrifuged at 12 000 *g* for 20 min at 4°C to collect the pellet containing crude mitochondria. Isolated mitochondria (1 mg) were resuspended in 200 μL of reaction mixture for incubation. The reaction mixture contained 500 μM pyruvate, or 500 μM malate, or a mixture of 500 μM ^13^C_3_‐labelled pyruvate and 500 μM malate, with the addition of 2 mM nicotinamide adenine dinucleotide (NAD^+^), 0.2 mM thiamine pyrophosphate (TPP), 0.012 mM coenzyme A (CoA) and 1 mM adenosine diphosphate (ADP). At the corresponding incubation time points, 0.5 M sucrose solution (pH = 1.0) for terminating the reaction was quickly added to the reaction mixture and mixed thoroughly. The substrate uptake by mitochondria was terminated by rapid centrifugation (12 000 *g*, 3 min). 20 μL of the resulting mitochondrial supernatant was collected for subsequent analysis.

### 2.5 Analysis of Tricarboxylic Acid (TCA) Cycle Metabolites

Citrate, pyruvate, malate, succinate, 2‐oxoglutarate and ^13^C_2_‐labelled citrate standards were purchased from Shanghai Yuanye Bio‐Technology Co. Ltd. ^13^C_3_‐labelled pyruvate was purchased from Beijing Solarbio Science and Technology Co. Ltd. Samples were analysed using a high‐performance liquid chromatography (HPLC, Agilent 1260) coupled with a linear ion trap triple quadrupole liquid chromatography‐tandem mass spectrometer (QTRAP5500; AB Sciex, USA) operated in multiple reaction monitoring (MRM) mode. Chromatographic separation was performed on a C18 column (Agilent Eclipse Plus, 100 mm × 2.1 mm, 3.5 μm) using a binary gradient elution with 0.1% formic acid in water (mobile phase A) and 0.1% formic acid in methanol (mobile phase B). The elution gradient was as follows: 18% B at 0 min, 18% B at 1 min, 34% B at 3 min, 100% B at 4 min, maintained at 100% B until 8 min, 18% B at 8.1 min and maintained at 18% B until 15 min. The column flow rate was 0.3 mL/min, the column temperature was 40°C, and the injection volume was 5 μL. Data were collected using *Analyst 1.6.1* software. Quantification of both isotopically labelled and unlabelled tricarboxylic acid (TCA) cycle metabolites was based on standard curves generated from standard substances.

### 2.6 Targeted Lipidomics Analysis

Qualitative and quantitative analysis of lipid molecules in S1 and S2 samples was performed using ultra‐performance liquid chromatography–tandem mass spectrometry (UPLC‐MS/MS) technology. Lipid extraction and specific analytical procedures were carried out according to the method previously reported (Wang, Yu, Liu, et al. 2025).

### 2.7 Data Analysis

Statistical significance of differences was analysed using the two‐tailed t‐test in *Excel 2016*, as well as one‐way analysis of variance (one‐way ANOVA) combined with Tukey's test in *IBM SPSS Statistics 26*.

## 3 Results

### 3.1 Time‐Series Transcriptome Reveals Enhanced Energy Metabolism During Thermogenesis

**FIGURE 1:**
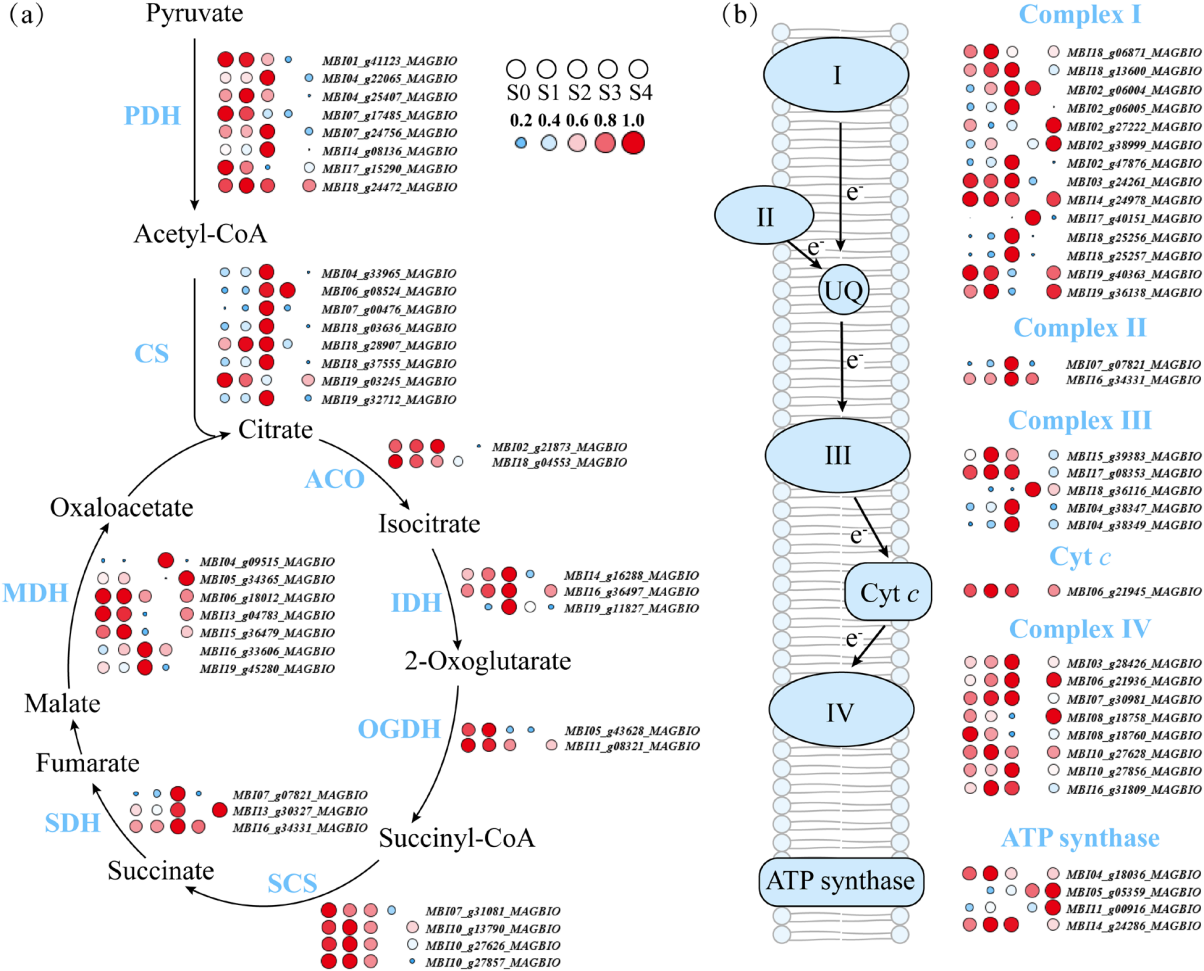
Temporal expression patterns of differentially expressed genes (DEGs) related to the tricarboxylic acid (TCA) cycle and oxidative phosphorylation (OXPHOS). (a) Temporal expression patterns of DEGs related to the TCA cycle. ACO, Aconitase hydratase; CS, Citrate synthase; IDH, Isocitrate dehydrogenase; MDH, Malate dehydrogenase; OGDH, 2‐oxoglutarate dehydrogenase; SCS, Succinyl‐CoA synthetase; SDH, Succinate dehydrogenase; PDH, Pyruvate dehydrogenase. (b) Temporal expression patterns of DEGs related to OXPHOS. I, NADH dehydrogenase; II, Succinate dehydrogenase; III, Cytochrome bc1 complex; IV, Cytochrome *c* oxidase; Cyt *c*, Cytochrome *c*; UQ, Ubiquinone. The colour and size of the circles represent the expression levels: Small blue circles indicate low expression, and large red circles indicate high expression. Expression values are presented as FPKM (Fragments Per Kilobase of transcript per Million mapped reads) normalised by the z‐score method.

The development of *M. denudata* flowers was divided into five stages (S0–S4): S0 represents the petal‐unexposed stage, S1 represents the pre‐thermogenic stage, S2 represents the thermogenic peak stage, S3 represents the post‐thermogenic peak stage and S4 represents the wilting stage (Figure S1). To investigate the dynamic changes in energy metabolism during thermogenesis in *M. denudata* , we employed time‐series transcriptome sequencing to analyse the expression patterns of genes related to the tricarboxylic acid (TCA) cycle and oxidative phosphorylation (OXPHOS) across the five developmental stages. A total of 37 differentially expressed genes (DEGs) associated with the TCA cycle were identified (Table S1), among which 29 showed higher expression levels during the thermogenic stage (S2). These included six genes encoding pyruvate dehydrogenase (PDH), seven genes encoding citrate synthase (CS), two genes encoding aconitase hydratase (ACO), three genes encoding isocitrate dehydrogenase (IDH), one gene encoding 2‐oxoglutarate dehydrogenase (OGDH), four genes encoding succinyl‐CoA synthetase (SCS), three genes encoding succinate dehydrogenase (SDH) and three genes encoding malate dehydrogenase (MDH) (Figure 1a).

Additionally, 34 DEGs related to OXPHOS were identified (Table S2). Eight out of 14 genes encoding Complex I were significantly upregulated during thermogenesis. All genes encoding Complex II showed significantly increased expression during thermogenesis, and four out of five genes encoding Complex III were significantly upregulated during thermogenesis. One gene encoding cytochrome *c* exhibited the highest expression levels in the pre‐thermogenic (S1) and thermogenic peak (S2) stages, and six out of eight genes encoding Complex IV showed higher expression during thermogenesis. Notably, among the four genes encoding ATP synthase, *MBI04_g18036_MAGBIO* and *MBI14_g24286_MAGBIO* had relatively high expression levels in petal‐unexposed (S0), pre‐thermogenic (S1) and the thermogenic peak (S2) stages, *MBI05_g05359_MAGBIO* was more highly expressed in post‐thermogenic peak (S3) and the wilting (S4) stages, while *MBI11_g00916_MAGBIO* was significantly downregulated during the thermogenic stage (S2) (Figure 1b). The downregulation of some ATP synthase genes suggests a potential shift from energy production (ATP) to heat production during thermogenesis. These results reflect the significantly active expression of genes involved in the TCA cycle and OXPHOS during the thermogenic stage, indicating that *M. denudata* flowers undergo enhanced energy metabolism processes during thermogenesis.

### 3.2 Time‐Series Transcriptome Reveals Enhanced Starch Hydrolysis and Glycolysis During Thermogenesis

**FIGURE 2:**
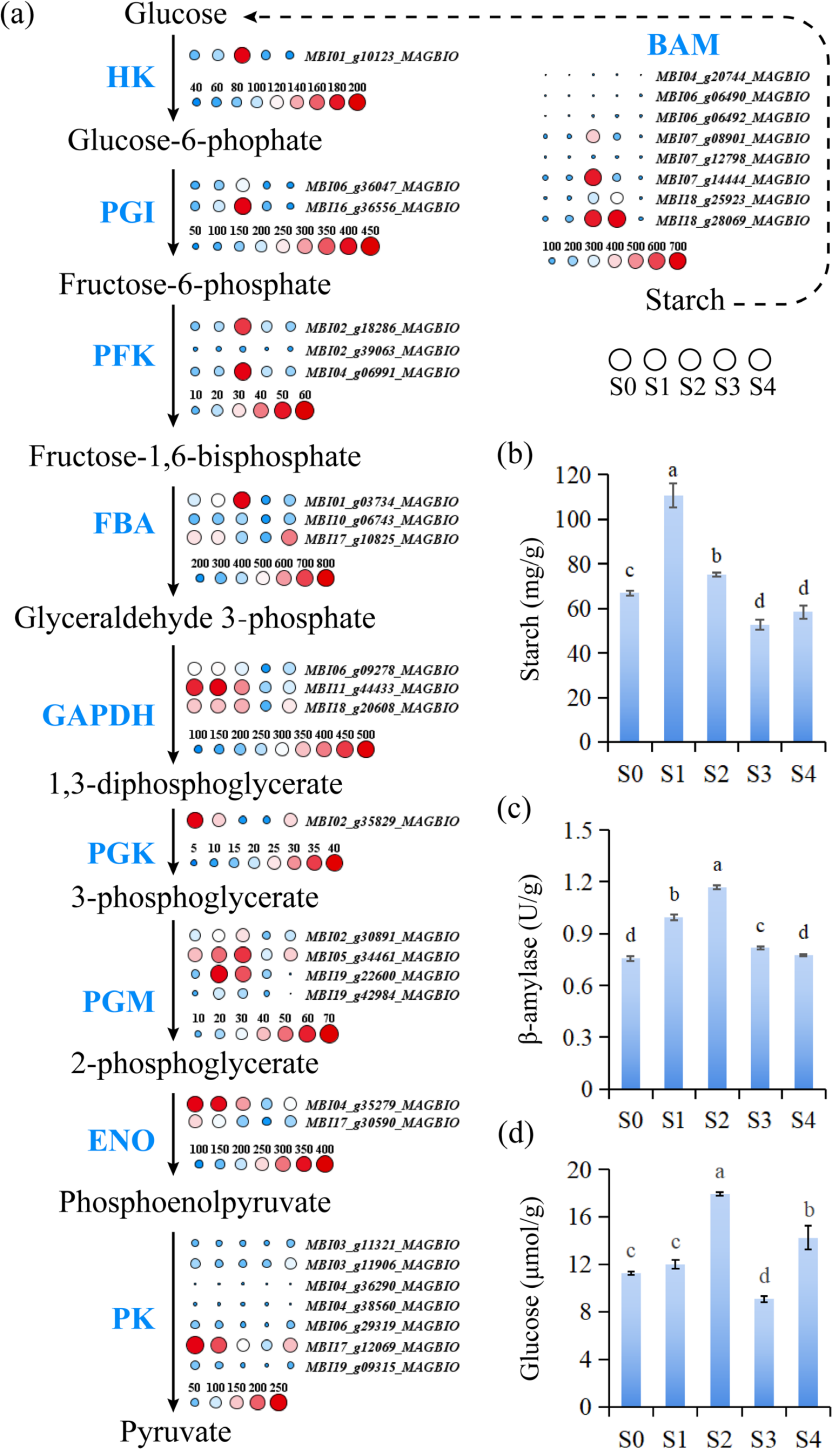
Enhanced starch hydrolysis and glycolysis during thermogenesis. (a) Temporal expression patterns of differentially expressed genes (DEGs) related to glycolysis. BAM, β‐amylase; ENO, Enolase; FBA, Fructose‐bisphosphate aldolase; HK, Hexokinase; GAPDH, Glyceraldehyde‐3‐phosphate dehydrogenase; PGI, Glucose‐6‐phosphate isomerase; PFK, 6‐phosphofructokinase; PGK, Phosphoglycerate kinase; PGM, Phosphoglyceromutase; PK, Pyruvate kinase. The colour and size of the circles represent the expression levels: Small blue circles indicate low expression, and large red circles indicate high expression. Expression values are presented as FPKM. (b) Changes in starch content at different developmental stages. (c) Changes in β‐amylase activity at different developmental stages. (d) Changes in glucose content at different developmental stages. Data are shown as mean ± SD (*n* = 3). Different lowercase letters indicate significant differences (*p* < 0.05, Tukey's test).

Given that carbohydrates are core substrates for plant energy metabolism, we further analysed the DEGs involved in starch hydrolysis and glycolysis, as well as the dynamic changes in carbohydrate contents. Time‐series transcriptome analysis revealed that all genes encoding β‐amylase were significantly upregulated during the thermogenic stage (S2) (Figure 2a). Among them, the expression levels of *MBI07_g08901_MAGBIO* and *MBI07_g14444_MAGBIO* in the thermogenic stage (S2) were more than nine‐fold higher than those in the pre‐thermogenic stage (S1) (Table S3), indicating enhanced starch hydrolysis during thermogenesis. A total of 26 DEGs related to glycolysis were identified (Figure 2a and Table S4). Among these, one gene encoding hexokinase (HK), two genes encoding glucose‐6‐phosphate isomerase (PGI) and three genes encoding 6‐phosphofructokinase (PFK) were significantly upregulated during the thermogenic stage (S2). Additionally, two out of three genes encoding fructose‐bisphosphate aldolase (FBA) showed the highest expression levels during the thermogenic stage. Furthermore, three genes encoding glyceraldehyde‐3‐phosphate dehydrogenase (GAPDH) exhibited higher expression levels in the pre‐thermogenic (S1) and thermogenic (S2) stages, and one gene encoding phosphoglycerate kinase (PGK) had higher expression levels in the pre‐thermogenic stage (S1). Four genes encoding phosphoglyceromutase (PGM) all showed higher expression during the thermogenic stage (S2), and two genes encoding enolase (ENO) displayed higher expression in the pre‐thermogenic (S1) and thermogenic (S2) stages. Among the seven genes encoding pyruvate kinase (PK), *MBI04_g38560_MAGBIO* had the highest expression level during the thermogenic stage (S2), *MBI17_g12069_MAGBIO* and *MBI19_g09315_MAGBIO* showed the highest expression in the pre‐thermogenic stage (S3) and the remaining genes exhibited distinct expression patterns.

Starch content increased significantly in the pre‐thermogenic stage (S1) but decreased significantly during the thermogenic stage (S2) (Figure 2b). Correspondingly, β‐amylase activity was the highest during the thermogenic stage (S2) (Figure 2c), which is consistent with the transcriptome results, indicating that starch stored in the pre‐thermogenic stage (S1) is utilised during thermogenesis. Glucose content was the highest during the thermogenic stage (S2) and then decreased significantly in the post‐thermogenic peak stage (S3) (Figure 2d), suggesting that glucose, as a core substrate, is rapidly metabolised through the glycolysis pathway to meet the high energy demand of the thermogenic process.

### 3.3 Pyruvate Imported Into the TCA Cycle via MPC Increased During Thermogenesis

**FIGURE 3:**
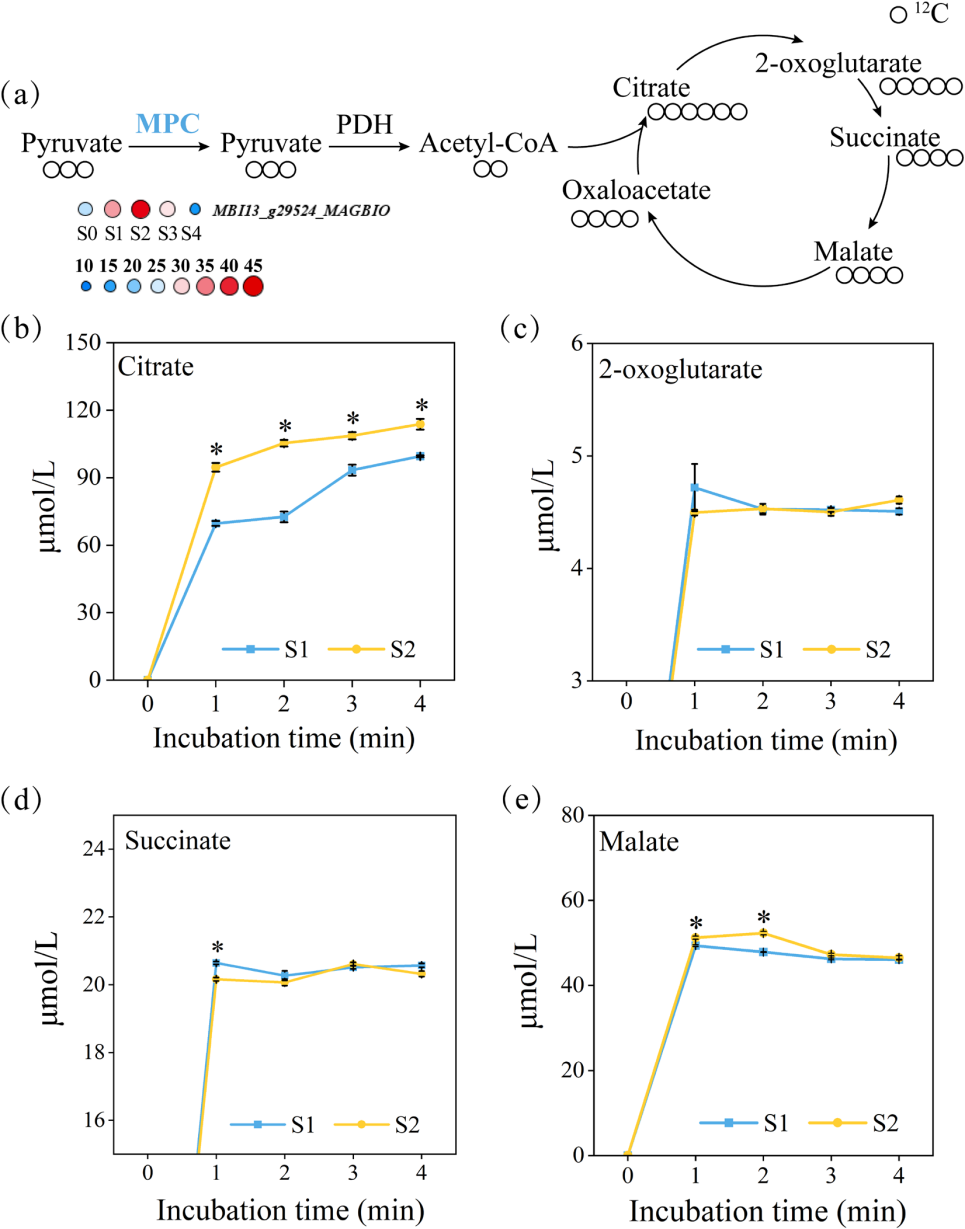
Contribution of MPC‐derived pyruvate to TCA cycle. (a) Schematic diagram showing the influx of MPC‐derived pyruvate into the TCA cycle. MPC, Mitochondrial pyruvate carrier; PDH, Pyruvate dehydrogenase. Changes in the content of unlabelled citrate (b), unlabelled 2‐oxoglutarate (c), unlabelled succinate (d) and unlabelled malate (e) in mitochondrial incubation medium after feeding with unlabelled pyruvate. Data are presented as mean ± SD (*n* = 3). *p < 0.05, two‐tailed Student's *t* test.

Since pyruvate generated from glycolysis enters mitochondria through mitochondrial pyruvate carriers (MPC) and then feeds into the TCA cycle, we analysed the expression pattern of MPC‐encoding genes. A differentially expressed MPC‐encoding gene, *MBI13_g29524_MAGBIO*, was identified (Table S5). Its expression gradually increased from the petal‐unexposed stage (S0) to the thermogenic stage (S2), reached the highest level during S2 and significantly decreased after thermogenesis (Figure 3a). To evaluate the contribution of pyruvate entering mitochondria via MPC to respiration, we isolated mitochondria from gynoecia at the thermogenic stage (S2) and pre‐thermogenic stage (S1) and supplied unlabelled pyruvate alone as the energy substrate. The relative amount of unlabelled citrate exported into the external mitochondrial medium was used to assess the flux of the TCA cycle driven by pyruvate derived from MPC. It was shown that mitochondria from S2 produced significantly more citrate than those from S1 within 1–4 min of incubation (Figure 3b), indicating that MPC‐mediated pyruvate import efficiency was significantly enhanced during thermogenesis to meet the increased energy demand of heat production. In contrast, the levels of 2‐oxoglutarate (2‐OG) showed no significant difference between the two stages within the same time period (Figure 3c), and statistically significant differences in succinate and malate contents were not detected after 4 min of incubation (Figure 3d,e). This suggests that the metabolic turnover rate of 2‐oxoglutarate, succinate and malate inside mitochondria is relatively fast during the thermogenic stage.

### 3.4 Pyruvate Generated by NAD‐ME Is Involved in Thermogenesis

**FIGURE 4:**
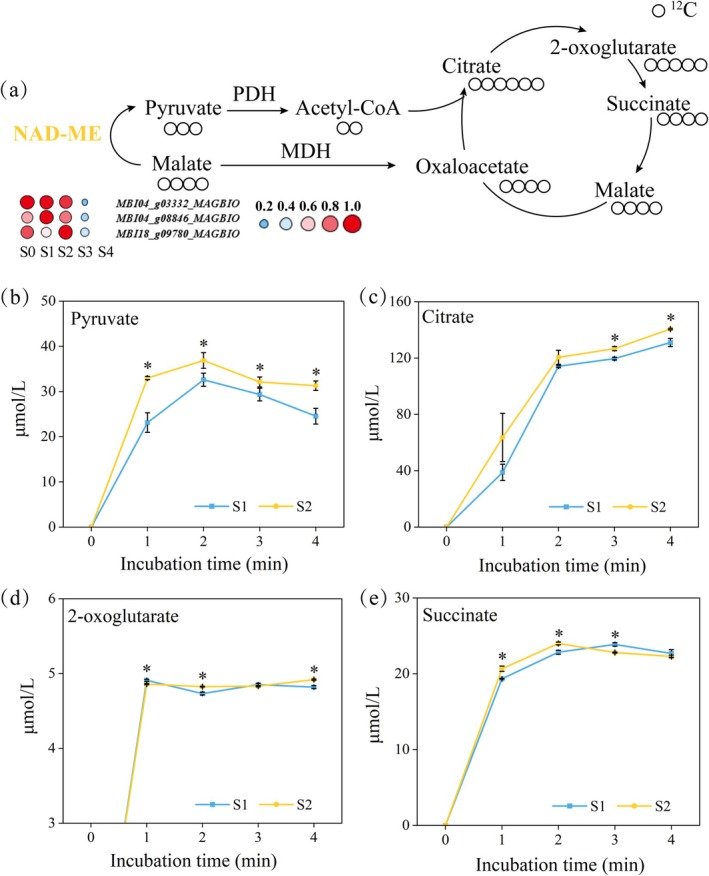
Contribution of NAD‐ME‐derived pyruvate to the TCA cycle. (a) Schematic diagram showing the influx of NAD‐ME‐derived pyruvate into the TCA cycle. NAD‐ME, NAD‐malic enzyme; MDH, Malate dehydrogenase; PDH, Pyruvate dehydrogenase. Changes in the content of unlabelled pyruvate (b), unlabelled citrate (c), unlabelled 2‐oxoglutarate (d) and unlabelled succinate (e) in mitochondrial incubation medium after feeding with unlabelled malate. Data are presented as mean ± SD (*n* = 3). *p < 0.05, two‐tailed Student's *t* test.

Due to the highly active energy metabolism in plant tissues during thermogenesis, the demand for respiratory substrates in the mitochondria of thermogenic cells increases significantly. Since NAD‐malic enzyme (NAD‐ME) can also mediate the conversion of malate to pyruvate, we further investigated whether the increased flux of pyruvate derived from NAD‐ME could serve as one of the substrate supply pathways during thermogenesis. Time‐series transcriptome analysis showed that the expression levels of NAD‐ME‐related genes in the pre‐thermogenic stages (S0, S1) and thermogenic peak stage (S2) were higher than those in the post‐thermogenic stages (S3, S4) (Figure 4a and Table S6). Subsequently, we fed mitochondria isolated from gynoecia at S1 and S2 with unlabelled malate. Malate can be converted to pyruvate by NAD‐ME and then enter the TCA cycle to support mitochondrial respiration. By measuring the levels of pyruvate, citrate, 2‐oxoglutarate and succinate in the external mitochondrial medium, we found that mitochondria from S2 produced significantly more pyruvate, citrate and 2‐oxoglutarate than those from S1 after 1–4 min of incubation (Figure 4b–e), indicating that the flux of pyruvate derived from NAD‐ME is upregulated during thermogenesis.

### 3.5 Both MPC‐ and NAD‐ME‐Derived Pyruvate Fluxes Increase During Thermogenesis

**FIGURE 5:**
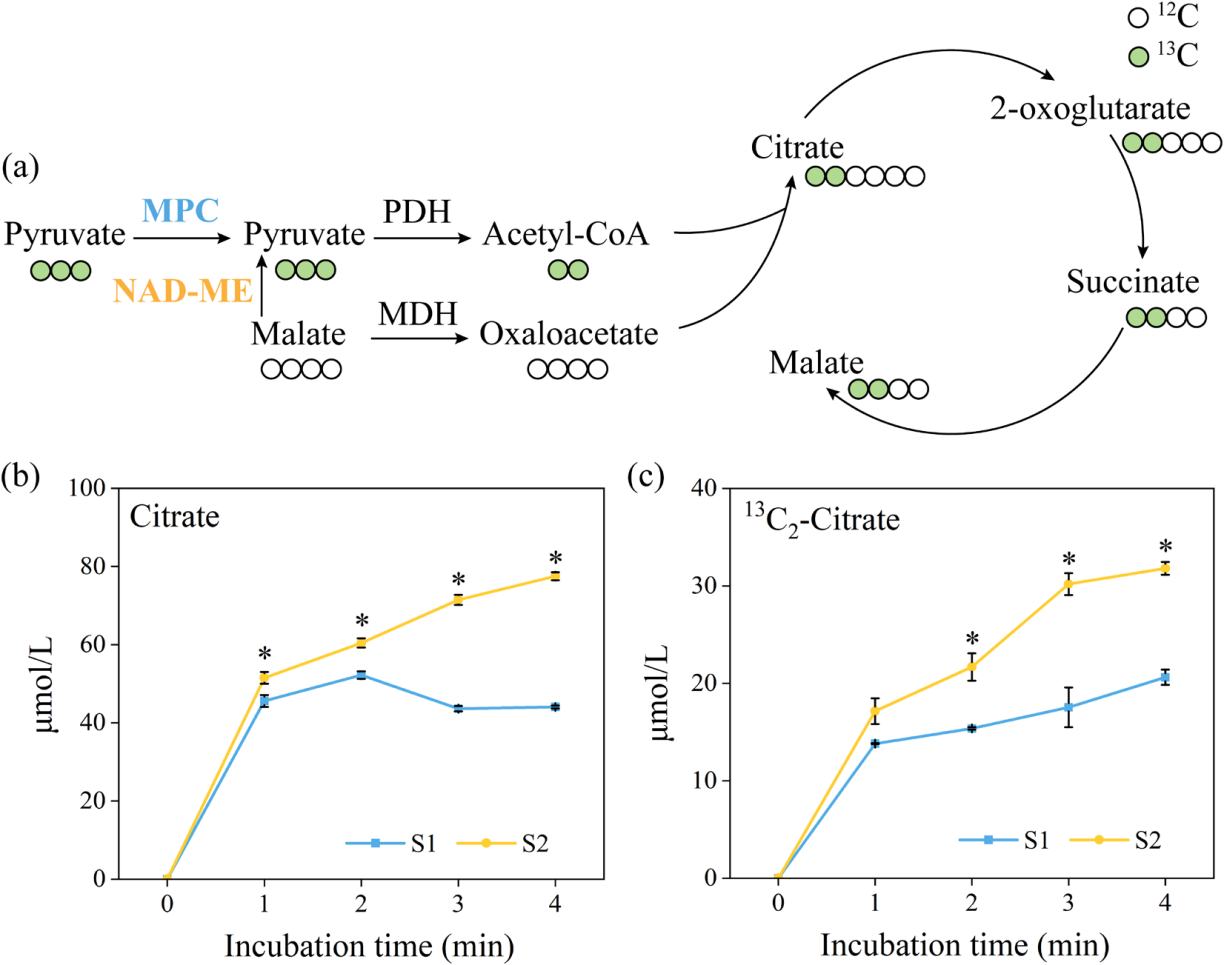
Contributions of MPC‐ and NAD‐ME‐derived pyruvate to the TCA cycle. (a) Schematic diagram showing the influx of labelled pyruvate into citrate. MDH, Malate dehydrogenase; MPC, Mitochondrial pyruvate carrier; NAD‐ME, NAD‐malic enzyme; PDH, Pyruvate dehydrogenase. Changes in the content of unlabelled citrate (b) and labelled citrate (c) in mitochondrial incubation medium after co‐feeding with labelled pyruvate and unlabelled malate. Data are presented as mean ± SD (*n* = 3). *p < 0.05, two‐tailed Student's *t* test.

Previous studies have shown that when the function of MPC is impaired or the supply of exogenous pyruvate is limited, the flux of pyruvate derived from NAD‐ME into the TCA cycle increases. However, under conditions where MPC function is normal and pyruvate supply is sufficient, NAD‐ME‐derived pyruvate tends to be preferentially exported out of mitochondria (Le et al. 2022). On the basis of this, it is necessary to clarify whether the increased flux of NAD‐ME‐derived pyruvate during the thermogenic stage (S2) is caused by insufficient supply via the MPC pathway. We isolated mitochondria from gynoecia at the pre‐thermogenic stage (S1) and thermogenic stage (S2) and co‐incubated them with labelled pyruvate and unlabelled malate at equal concentrations. Labelled pyruvate enters the mitochondrial matrix through MPC, is converted to labelled acetyl‐CoA by pyruvate dehydrogenase (PDH) and then condenses with unlabelled oxaloacetate via citrate synthase (CS) to generate labelled citrate (Figure 5a). Meanwhile, unlabelled malate can be converted to unlabelled pyruvate by NAD‐ME, and the latter is further metabolised to unlabelled acetyl‐CoA, which then condenses with unlabelled oxaloacetate to form unlabelled citrate (Figure 5a).

The feeding results showed that after 4 min of incubation, the levels of both labelled and unlabelled citrate in the external mitochondrial medium were significantly higher in S2 than in S1 (Figure 5b,c). Interestingly, the content of unlabelled citrate was consistently higher than that of labelled citrate within the 4‐min incubation period, indicating that citrate is more derived from pyruvate converted by NAD‐ME rather than pyruvate transported by MPC. These results suggest that on the basis of sufficient pyruvate supply dominated by MPC, the flux of NAD‐ME‐derived pyruvate is also enhanced, and NAD‐ME‐derived pyruvate may contribute more. This forms a dual‐pathway synergistic energy supply mechanism, providing metabolic flexibility to meet the high energy demand of the thermogenic process.

### 3.6 Ability to Transport Substrates Into Mitochondria by β‐Oxidation Enhanced During Thermogenesis

**FIGURE 6:**
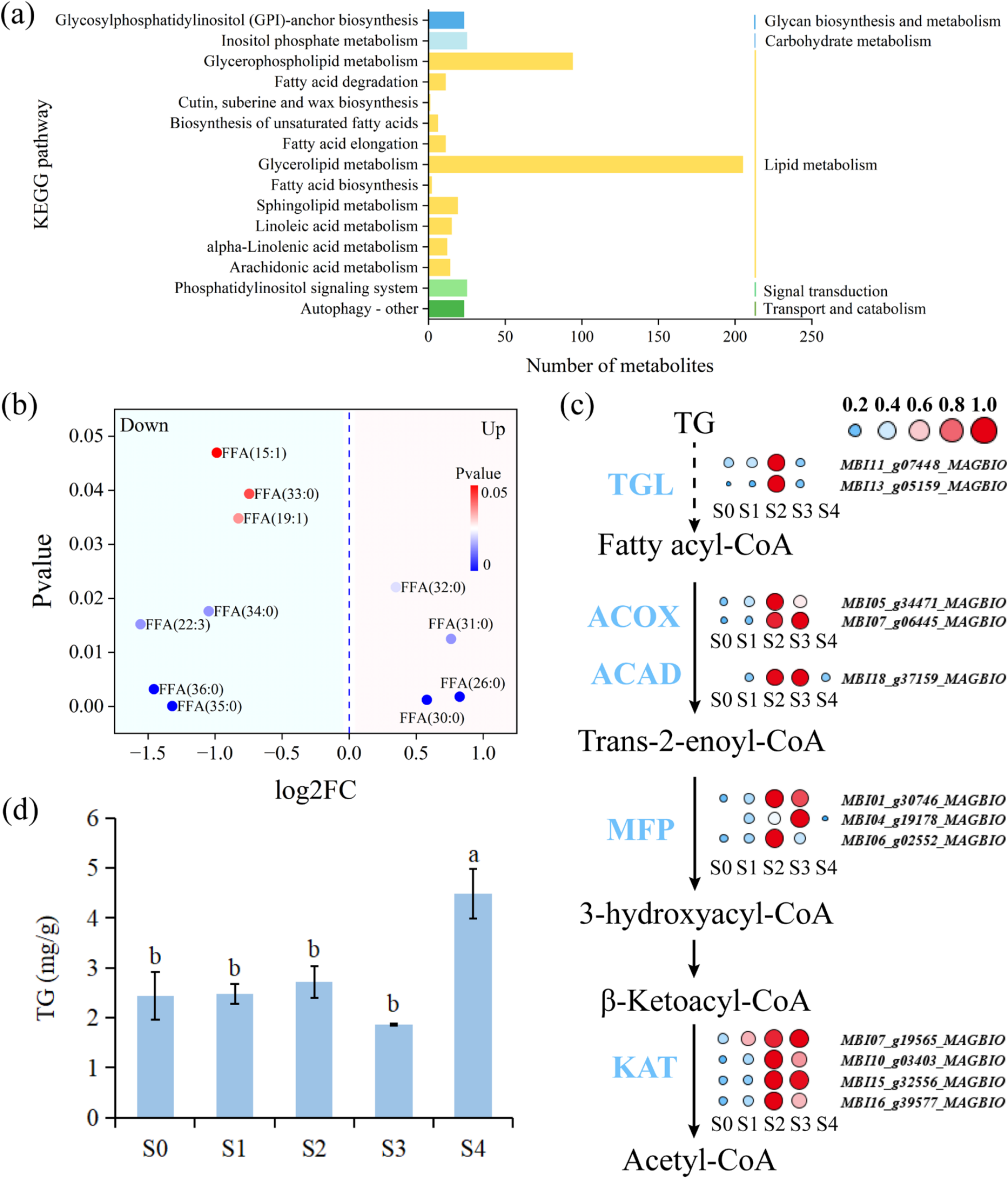
Contribution of fatty acid β‐oxidation to thermogenesis. (a) KEGG enrichment pathway analysis between pre‐thermogenic (S1) and thermogenic (S2) stages. (b) Changes in differential metabolites related to fatty acid degradation. (c) Temporal expression patterns of differentially expressed genes (DEGs) associated with fatty acid β‐oxidation. ACAD, Acyl‐CoA dehydrogenase; ACOX, Acyl‐CoA oxidase; KAT, 3‐ketoacyl‐CoA thiolase; MFP, Multifunctional protein; TGL, Triacylglycerol lipase. The colour and size of the circles represent the expression levels: Small blue circles indicate low expression, and large red circles indicate high expression. Expression levels are presented as FPKM values normalised by the z‐score method. (d) Changes in triglyceride content at different developmental stages. Data are shown as mean ± SD (*n* = 3). Different lowercase letters indicate significant differences (*p* < 0.05, Tukey's test).

Fatty acid β‐oxidation is the core metabolic pathway for intracellular fatty acid breakdown to obtain energy. Given the high demand for energy substrates during thermogenesis, whether fatty acids can also provide energy for thermogenesis deserves further investigation. Firstly, we examined changes in lipid metabolites in gynoecia at the pre‐thermogenic stage (S1) and thermogenic stage (S2), and a total of 313 differential lipid metabolites were detected (Table S7). Hierarchical cluster analysis, principal component analysis (PCA) and orthogonal partial least squares discriminant analysis (OPLS‐DA) showed clear separation between S1 and S2 samples, with 257 lipids exhibiting decreased content during thermogenesis (Figure S2). KEGG enrichment analysis revealed that these 313 differential metabolites were enriched in 15 metabolic pathways, including the fatty acid degradation pathway (ko00071) (Figure 6a). Further analysis of 11 differential metabolites related to fatty acid degradation pathways showed that 63.6% of fatty acids were significantly reduced during the thermogenic stage (S2) (Figure 6b), indicating enhanced fatty acid catabolism during thermogenesis.

We further explored DEGs related to fatty acid β‐oxidation through time‐series transcriptome analysis. It was shown that two genes encoding triacylglycerol lipase (TGL) were significantly upregulated during the thermogenic stage (S2) (Figure 6c and Table S8), which can catalyse the hydrolysis of triacylglycerol to release fatty acids (Fan et al. 2017). Additionally, during the thermogenic stage (S2), the expression levels of 10 DEGs encoding key enzymes in the fatty acid β‐oxidation pathway – including acyl‐CoA oxidase (ACOX), acyl‐CoA dehydrogenase (ACAD), multifunctional protein (MFP) and 3‐ketoacyl‐CoA thiolase (KAT) – were significantly increased (Figure 6c and Table S9). This further supports the possibility of fatty acid β‐oxidation as an energy supply pathway in thermogenic tissues. Furthermore, we found that triglyceride (TG) content remained low throughout the petal‐unexposed and post‐thermogenic peak stages (S0–S3) but increased significantly during the wilting stage (S4) (Figure 6d), indicating that TG is rapidly decomposed during the thermogenic stage to provide more fatty acids as an energy source.

### 3.7 Ability to Transport Substrates Into Mitochondria From Alanine and Glutamate Enhanced During Thermogenesis

**FIGURE 7:**
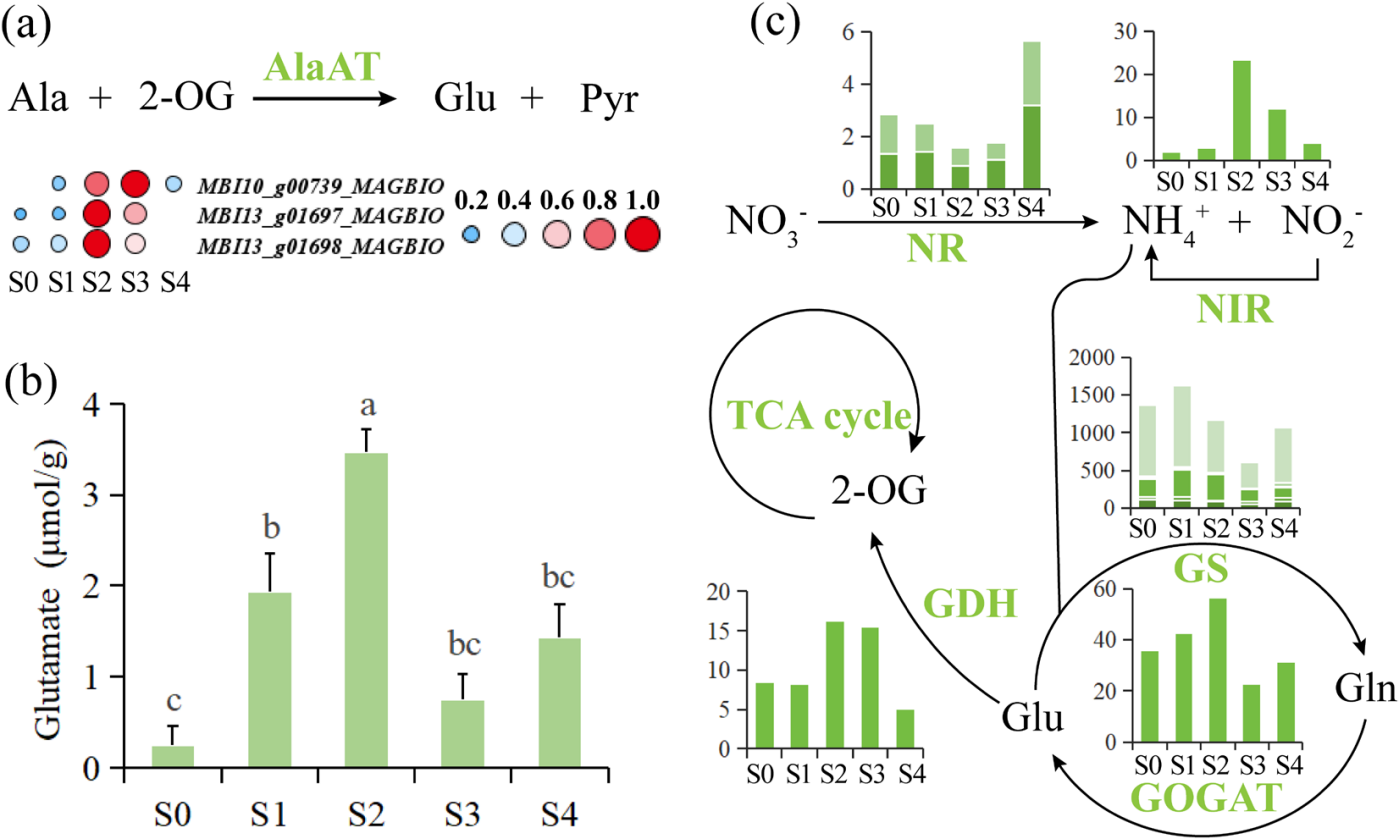
Contribution of alanine and glutamate metabolism to thermogenesis. (a) Expression dynamics of differential genes encoding alanine aminotransferase (AlaAT) across different developmental stages. 2‐OG, 2‐oxoglutarate; Ala, Alanine; Glu, Glutamate; Pyr, Pyruvate. The colour and size of the circles represent the expression levels: Small blue circles indicate low expression, and large red circles indicate high expression. Expression levels are presented as FPKM values normalised by the z‐score method. (b) Changes in glutamate content across five developmental stages. Data are shown as mean ± SD (*n* = 3). Different lowercase letters indicate significant differences (*p* < 0.05, Tukey's test). (c) Expression profiles of differential genes related to nitrogen metabolism. GDH, Glutamate dehydrogenase; Gln, Glutamine; GOGAT, Glutamate synthase; GS, Glutamine synthetase; NIR, Nitrite reductase; NR, Nitrate reductase. The vertical axis represents FPKM values.

Pyruvate generated from alanine via alanine aminotransferase (AlaAT) is also an important respiratory substrate in plants. Time‐series transcriptome analysis showed that all differentially expressed AlaAT‐related genes were significantly upregulated during the thermogenic stage (S2) (Figure 7a and Table S10), indicating that pyruvate transported into mitochondria via the AlaAT pathway may serve as a crucial respiratory substrate. Moreover, the generated glutamate can be converted to 2‐oxoglutarate by glutamate dehydrogenase (GDH), thereby entering the TCA cycle. By measuring glutamate content across various developmental stages, we found that glutamate content was significantly higher during the thermogenic stage (S2) compared to other stages (Figure 7b), which also provides evidence for the enhancement of the AlaAT pathway during thermogenesis. After plants absorb nitrate (NO_3_^−^), it can be reduced to nitrite (NO_2_^−^) by nitrate reductase (NR), and subsequently further reduced to ammonium (NH_4_^+^) by nitrite reductase (NIR). Finally, glutamate is synthesised via the glutamine synthetase (GS)‐glutamate synthase (GOGAT) pathway (Liu et al. 2024). The generated glutamate can enter mitochondria and be converted to 2‐oxoglutarate by GDH, providing intermediates for the TCA cycle (Liao et al. 2022).

Therefore, we analysed the expression patterns of DEGs related to nitrogen metabolism across different developmental stages (Table S11). It was shown that genes encoding nitrate reductase (NR) were significantly downregulated during the thermogenic stage (S2), while genes related to nitrite reductase (NIR) encoding were significantly upregulated. The expression of glutamine synthetase (GS)‐related genes was downregulated at stage S2, whereas the expression of glutamate synthase (GOGAT)‐related genes peaked at this stage, indicating enhanced glutamate biosynthesis during S2, which is consistent with the highest glutamate content observed at S2. Additionally, the expression of glutamate dehydrogenase (GDH)‐related genes was significantly upregulated during the peak thermogenic stage (S2) (Figure 7c), suggesting an increased conversion of glutamate to 2‐oxoglutarate during thermogenesis, which also provides evidence that glutamate may serve as an energy substrate during thermogenesis.

## 4 Discussion

### 4.1 Floral Thermogenesis Is Accompanied by Enhanced Respiratory Metabolism

Plant thermogenesis is closely associated with intense mitochondrial respiratory metabolism (Barreto et al. 2025). Early studies have shown that total respiratory flux increases during receptacle thermogenesis in lotus (Grant et al. 2008). In this study, core energy metabolic pathways such as the TCA cycle and oxidative phosphorylation were significantly upregulated during the thermogenic stage of *M. denudata* . Interestingly, 50% of ATP synthase‐encoding genes exhibited lower expression during the thermogenic stage, suggesting a potential shift in energy distribution within the mitochondrial electron transport chain – specifically, a shift from ATP synthesis to heat release. This is consistent with the high expression of alternative oxidase (AOX) during thermogenesis in *M. denudata* and lotus (Wang et al. 2022; Yu, Wang, Kong, et al. 2025). AOX can bypass the phosphorylation sites of the electron transport chain, directly transferring electrons to oxygen and releasing energy as heat (Van Aken et al. 2009). Meanwhile, β‐amylase activity and starch degradation were synchronously and significantly enhanced during thermogenesis, and genes encoding key glycolysis enzymes were upregulated during this stage (Figure 2). Furthermore, glucose content surged during thermogenesis, followed by rapid consumption, indicating upregulation of the glycolysis pathway during thermogenesis. Notably, starch accumulation in *M. denudata* flowers before thermogenesis and its rapid consumption during the thermogenic stage exhibit a transition from metabolic reserve to explosive release. This spatiotemporal regulation may be achieved through the temporal activation of β‐amylase genes by transcription factors (Zhang et al. 2022), providing a substrate basis for the short‐term heat burst.

### 4.2 Pyruvate From Different Sources Collectively Supports Thermogenesis

In addition to the upregulation of energy metabolic pathways, thermogenesis also relies on the flexible supply of substrates. Pyruvate is not only a major carbon source for mitochondrial respiratory metabolism but also can activate the activity of AOX in plant mitochondria (Millar et al. 1993; Selinski et al. 2016), which further drives the thermogenic process. The mitochondrial pyruvate carrier (MPC) is a protein complex located in the inner mitochondrial membrane, responsible for transporting pyruvate from outside the mitochondria to the mitochondrial matrix to generate acetyl‐CoA for the TCA cycle (He et al. 2019). Our study found that the transcriptional levels of MPC‐related genes increased significantly during the thermogenic stage, and mitochondrial feeding experiments confirmed that mitochondria isolated from thermogenic tissues had a higher pyruvate transport flux mediated by MPC.

NAD‐ME is a key enzyme regulating malate metabolism, which can catalyse the production of pyruvate from malate for entry into the TCA cycle. Typically, NAD‐ME‐mediated pyruvate only functions under specific conditions (e.g., when MPC function is deficient) (Le et al. 2021). In this study, the expression levels of NAD‐ME‐related genes in pre‐thermogenic stages (S0, S1) and thermogenic stage (S2) were higher than those in post‐thermogenic stages (S3, S4). Moreover, in vitro feeding experiments showed that the flux of pyruvate derived from NAD‐ME at S2 was significantly higher than that at S1. By further co‐feeding mitochondria with labelled pyruvate and unlabelled malate, we found that when pyruvate derived from MPC was sufficient, the flux of NAD‐ME‐derived pyruvate also increased significantly during the thermogenic stage (S2). Additionally, when both pathways acted together, the contribution of the NAD‐ME pathway to citrate production was greater than that of MPC. This finding breaks through the traditional understanding that NAD‐ME in *A. thaliana* only acts as an ‘emergency valve’ (Le et al. 2021), revealing a unique regulatory pattern of pyruvate supply in thermogenic *M. denudata* . This dual‐pathway synergistic pattern not only ensures basic energy supply but also endows the metabolic system with the flexibility to cope with high energy‐consuming states.

### 4.3 Lipid Metabolism and β‐Oxidation Are Active During Thermogenesis

The active lipid metabolism during thermogenesis is an important manifestation of the dynamic regulation of the energy metabolic network. Early studies found that the respiratory exchange ratio of the thermogenic plant *Philodendron selloum* during thermogenesis was 0.83 (Seymour et al. 1984), and the ratio of ^13^C–^12^C in the carbon dioxide produced by respiration decreased (Walker et al. 1983), directly indicating that it mainly uses lipids as respiratory substrates during thermogenesis. In this study, the content of triglyceride (TG) was low during thermogenesis, while the transcriptional level of TGL, a key enzyme for TG hydrolysis, was significantly upregulated during the thermogenic stage. Meanwhile, 63.6% of fatty acids showed a significant decrease in content during this thermogenic stage. Furthermore, genes encoding key enzymes in the fatty acid β‐oxidation pathway were all highly expressed during the thermogenic stage. These enzymes can catalyse the decomposition of fatty acids into acetyl‐CoA, which then merges into the TCA cycle metabolic flow (Li‐Beisson et al. 2013; Xiang et al. 2023). It has been reported that FFA can activate uncoupling proteins (UCP) in plant mitochondria (Sluse et al. 1998; Zhu et al. 2011). UCP can further mediate the transmembrane inflow of H^+^, uncoupling respiration from ATP synthesis, so that energy is released in the form of heat rather than converted into ATP (Vercesi et al. 2006). These results reveal that fatty acids may participate in the thermogenic process of *M. denudata* as respiratory substrates.

### 4.4 Nitrogen and Carbon Metabolism Are Coupled to Cope With the High Energy‐Demanding Thermogenic Process

Besides pyruvate generation mediated by MPC and NAD‐ME, the transamination reaction catalysed by alanine aminotransferase (AlaAT) constitutes a third pathway for pyruvate production (Lin et al. 2025). This enzyme can convert alanine and 2‐oxoglutarate into pyruvate and glutamate, while participating in both nitrogen and carbon metabolism (Miyashita et al. 2007). In this study, the transcriptional levels of AlaAT‐related genes were significantly upregulated during the thermogenic stage (S2), and glutamate content also peaked during this stage. These results indicate that the AlaAT pathway may be activated during thermogenesis, enhancing pyruvate supply and glutamate accumulation to supplement substrate provision for respiratory flux. The reaction catalysed by nitrate reductase (NR) consumes NAD(P)H, a coenzyme that also serves as an important reducing equivalent in energy‐producing pathways such as glycolysis and the TCA cycle (Campbell 2001; Xiao et al. 2018). In this study, the expression of genes encoding nitrite reductase (NIR) in *M. denudata* was significantly upregulated during the thermogenic stage, while NR gene expression was downregulated, likely forming a ‘NR inhibition‐NIR activation’ metabolic reprogramming. Specifically, reduced NR activity decreases NAD(P)H consumption in NO_3_^−^ reduction, avoiding competition for reducing equivalents with energy‐producing pathways like glycolysis and the TCA cycle, thereby allowing more reducing equivalents to be used for mitochondrial respiration. Upregulated NIR transcription accelerates the conversion of NO_2_^−^ to NH_4_^+^, while downregulated glutamine synthetase (GS) expression and upregulated glutamate synthase (GOGAT) expression promote glutamate synthesis via the GS‐GOGAT pathway (Zayed et al. 2023). Furthermore, enhanced expression of glutamate dehydrogenase (GDH) indicates that during thermogenesis, substrate supply derived from amino acids is more inclined toward GDH‐mediated conversion of glutamate to 2‐oxoglutarate, providing additional intermediates for the TCA cycle. This coupling of nitrogen metabolism and energy metabolism may represent an important adaptive strategy for thermogenic plants to cope with high energy demand.

**FIGURE 8:**
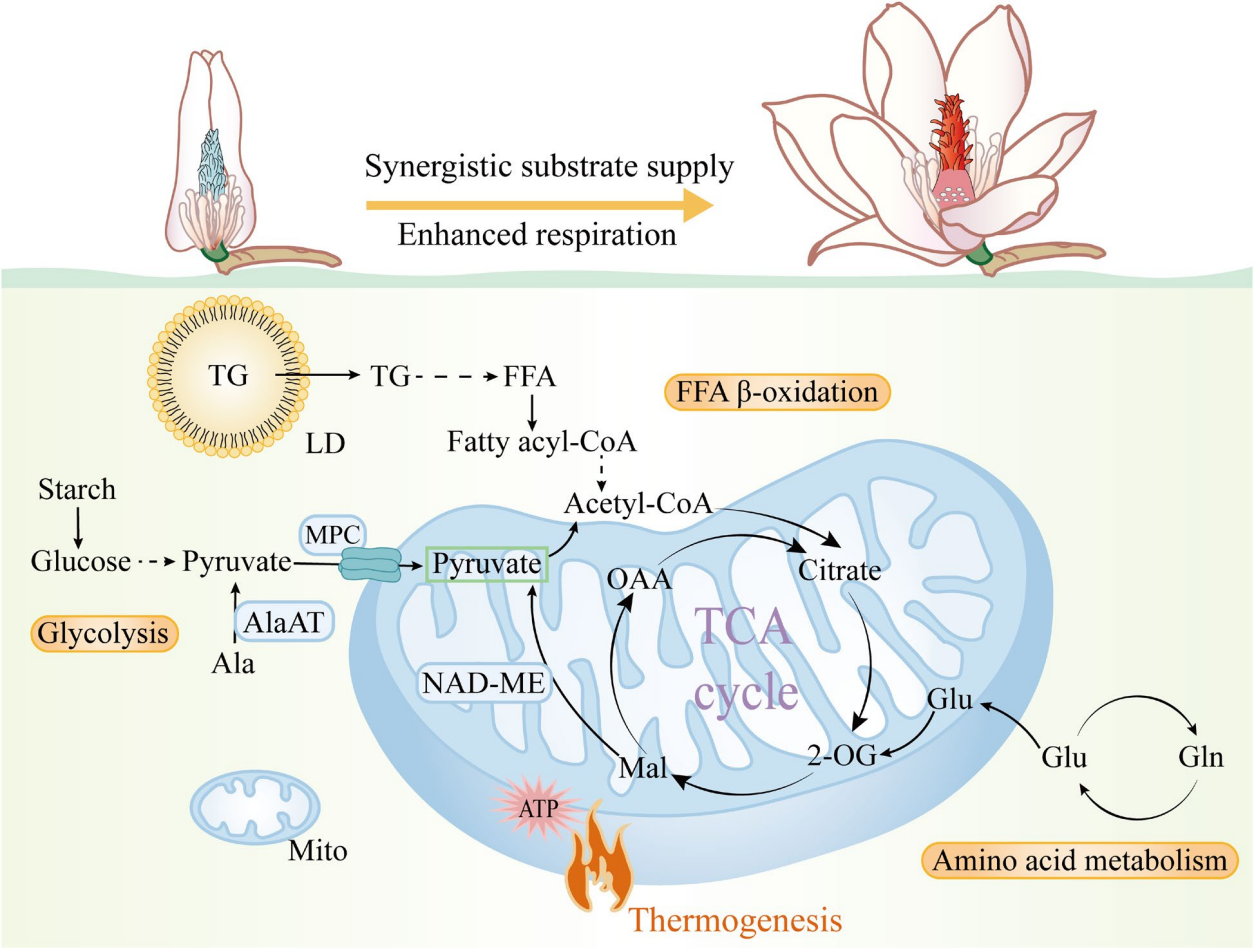
Synergistic supply mode of multi‐source substrates during floral thermogenesis in *M. denudata* . Starch is rapidly mobilised and hydrolysed into glucose; glucose is converted into pyruvate (pyr) via glycolysis, The latter enters the mitochondrial matrix through the mitochondrial pyruvate carrier (MPC). Alanine aminotransferase (AlaAT) mediates the conversion of alanine into pyruvate. NAD‐dependent malic enzyme (NAD‐ME) catalyses the decarboxylation of malate to generate pyruvate in the mitochondrial matrix. Pyruvate from these three sources enters the tricarboxylic acid (TCA) cycle. Triglycerides stored in lipid droplets (LDs) are hydrolysed into fatty acids, which generate acetyl‐CoA via β‐oxidation and enter the TCA cycle. In addition, glutamate is catalysed by glutamate dehydrogenase (GDH) to be converted into 2‐oxoglutarate, which participates in the TCA cycle. 2‐OG, 2‐oxoglutarate; Ala, Alanine; FFA, Free fatty acid; Gln, Glutamine; Glu, Glutamate; Mito, Mitochondrion; OAA, Oxaloacetate; Mal, Malate; TG, Triglyceride.

In summary, during floral thermogenesis, plants need to release a large amount of heat in a short time, which places extremely high demands on the supply of energy substrates (Figure 8). As a key intermediate product of cellular respiration, pyruvate can enter mitochondria through multiple pathways such as mitochondrial pyruvate carrier (MPC), NAD‐malic enzyme (NAD‐ME) and alanine aminotransferase (AlaAT) to participate in the TCA cycle. In addition, fatty acids are degraded via β‐oxidation to generate acetyl‐CoA, and amino acids are converted into TCA cycle intermediates through deamination, both of which may provide energy for the thermogenic process. Our study not only reveals the important role of glucose metabolism in thermogenesis but also preliminarily explores the potential contributions of fatty acid β‐oxidation and amino acid metabolism during the thermogenic stage. Substrates from multiple sources jointly support the thermogenic process, providing a new perspective for further understanding the metabolic mechanism underlying the massive heat release within a short period in plants.

## Author Contributions

R.W. and S.W. conceived and designed the project. S.W., J.L., Z.W., M.Y., C.L. and D.L. generated the experimental data. S.W. analysed the experimental data and wrote the manuscript. R.W. revised the manuscript. All authors critically reviewed and approved the final manuscript.

## Funding

This work was supported by National Natural Science Foundation of China, No. 32370391, Fundamental Research Funds for the Central Universities, No. 2025XJ06.

## Conflicts of Interest

The authors declare no conflicts of interest.

## Supporting information


**Figure S1:** Development and thermogenesis dynamics of the *M. denudata* flowers.
**Figure S2:**. Global analysis of lipid changes from S1 (the pre‐thermogenic stage) to S2 (the thermogenic peak stage) in *M. denudata* flowers.

**Table S1:** Differentially expressed genes (DEGs) related to tricarboxylic acid (TCA) cycle.
**Table S2:** Differentially expressed genes (DEGs) related to oxidative phosphorylation.
**Table S3:** Differentially expressed genes (DEGs) related to β‐amylase.
**Table S4:** Differentially expressed genes (DEGs) related to glucolysis.
**Table S5:** Differentially expressed genes (DEGs) related to mitochondrial pyruvate carrier (MPC).
**Table S6:** Differentially expressed genes (DEGs) related to NAD‐dependent malic enzyme (NAD‐ME).
**Table S7:** Profiles of differential lipid metabolites between S1 and S2.
**Table S8:** Differentially expressed genes (DEGs) related to triacylglycerol lipase.
**Table S9:** Differentially expressed genes (DEGs) related to fatty acid β‐oxidation.
**Table S10:** Differentially expressed genes (DEGs) related to alanine aminotransferase (AlaAT).
**Table S11:** Differentially expressed genes (DEGs) related to nitrogen metabolism.

## Data Availability

The data underlying this article is available in the article and in its Supporting Information.

## References

[pbi70479-bib-0001] Barreto, P. , E. Feitosa‐Araujo , A. R. Fernie , and M. Schwarzländer . 2025. “How to Turbo Charge Respiration – Thermogenic Metabolism in Plants.” Current Opinion in Plant Biology 85: 102730. 40311169 10.1016/j.pbi.2025.102730

[pbi70479-bib-0002] Bian, F. , Y. Luo , L. Li , Y. Pang , and Y. Peng . 2021. “Inflorescence Development, Thermogenesis and Flower‐Visiting Insect Activity in *Alocasia odora* .” Flora 279: 151818.

[pbi70479-bib-0003] Campbell, W. H. 2001. “Structure and Function of Eukaryotic NAD(P)H:Nitrate Reductase.” Cellular and Molecular Life Sciences: CMLS 58: 194–204. 11289301 10.1007/PL00000847 PMC11146515

[pbi70479-bib-0004] Dong, S. , M. Liu , Y. Liu , et al. 2021. “The Genome of *Magnolia biondii* Pamp. Provides Insights Into the Evolution of Magnoliales and Biosynthesis of Terpenoids.” Horticultural Research 8: 38. 10.1038/s41438-021-00471-9 PMC7917104 33642574

[pbi70479-bib-0005] Ducat, D. C. 2015. “Metabolic Engineering: Kick‐Starting TCA Cycling.” Nature Plants 1: 15058.

[pbi70479-bib-0006] Fan, J. , L. Yu , and C. Xu . 2017. “A Central Role for Triacylglycerol in Membrane Lipid Breakdown, Fatty Acid β‐Oxidation, and Plant Survival Under Extended Darkness.” Plant Physiology 174: 1517–1530. 28572457 10.1104/pp.17.00653 PMC5490926

[pbi70479-bib-0007] Fernie, A. R. , F. Carrari , and L. J. Sweetlove . 2004. “Respiratory Metabolism: Glycolysis, the TCA Cycle and Mitochondrial Electron Transport.” Current Opinion in Plant Biology 7: 254–261. 15134745 10.1016/j.pbi.2004.03.007

[pbi70479-bib-0008] Grant, N. M. , R. E. Miller , J. R. Watling , and S. A. Robinson . 2008. “Synchronicity of Thermogenic Activity, Alternative Pathway Respiratory Flux, AOX Protein Content, and Carbohydrates in Receptacle Tissues of Sacred Lotus During Floral Development.” Journal of Experimental Botany 59: 705–714. 18252702 10.1093/jxb/erm333

[pbi70479-bib-0009] He, L. , Y. Jing , J. Shen , et al. 2019. “Mitochondrial Pyruvate Carriers Prevent Cadmium Toxicity by Sustaining the TCA Cycle and Glutathione Synthesis.” Plant Physiology 180: 198–211. 30770461 10.1104/pp.18.01610 PMC6501077

[pbi70479-bib-0010] Jallet, D. , D. Xing , A. Hughes , et al. 2020. “Mitochondrial Fatty Acid β‐Oxidation Is Required for Storage‐Lipid Catabolism in a Marine Diatom.” New Phytologist 228: 946–958. 32535932 10.1111/nph.16744

[pbi70479-bib-0011] Kim, D. , J. M. Paggi , C. Park , C. Bennett , and S. L. Salzberg . 2019. “Graph‐Based Genome Alignment and Genotyping With HISAT2 and HISAT‐Genotype.” Nature Biotechnology 37: 907–915. 10.1038/s41587-019-0201-4 PMC7605509 31375807

[pbi70479-bib-0012] Le, X. H. , C.‐P. Lee , and A. H. Millar . 2021. “The Mitochondrial Pyruvate Carrier (MPC) Complex Mediates One of Three Pyruvate‐Supplying Pathways That Sustain Arabidopsis Respiratory Metabolism.” Plant Cell 33: 2776–2793. 34137858 10.1093/plcell/koab148 PMC8408480

[pbi70479-bib-0013] Le, X. H. , C. P. Lee , D. Monachello , and A. H. Millar . 2022. “Metabolic Evidence for Distinct Pyruvate Pools Inside Plant Mitochondria.” Nature Plants 8: 694–705. 35681019 10.1038/s41477-022-01165-3

[pbi70479-bib-0014] Lee, H. J. , J. Son , S. J. Sim , and H. M. Woo . 2020. “Metabolic Rewiring of Synthetic Pyruvate Dehydrogenase Bypasses for Acetone Production in Cyanobacteria.” Plant Biotechnology Journal 18: 1860–1868. 31960579 10.1111/pbi.13342 PMC7415776

[pbi70479-bib-0015] Liao, H. S. , Y. H. Chung , and M. H. Hsieh . 2022. “Glutamate: A Multifunctional Amino Acid in Plants.” Plant Science 318: 111238. 35351313 10.1016/j.plantsci.2022.111238

[pbi70479-bib-0016] Li‐Beisson, Y. , B. Shorrosh , F. Beisson , et al. 2013. “Acyl‐Lipid Metabolism.” Arabidopsis Book 11: e0161. 23505340 10.1199/tab.0161 PMC3563272

[pbi70479-bib-0017] Lin, K. , L. Yue , L. Yuan , et al. 2025. “Alanine Metabolism Mediates Energy Allocation of the Brown Planthopper to Adapt to Resistant Rice.” Journal of Advanced Research 67: 25–41. 38246245 10.1016/j.jare.2024.01.022 PMC11725158

[pbi70479-bib-0018] Liu, J. , L. Tong , X. Zhang , et al. 2024. “Dynamic Nitrogen Reallocation in Rice Plants Upon Insect Herbivory by a Generalist Lepidopteran Pest *Spodoptera litura* (Fabricius).” Plant, Cell & Environment 47, no. 1: 294–307. 10.1111/pce.14736 37843127

[pbi70479-bib-0019] Love, M. I. , W. Huber , and S. Anders . 2014. “Moderated Estimation of Fold Change and Dispersion for RNA‐Seq Data With DESeq2.” Genome Biology 15: 550. 25516281 10.1186/s13059-014-0550-8 PMC4302049

[pbi70479-bib-0020] Millar, A. H. , J. T. Wiskich , J. Whelan , and D. A. Day . 1993. “Organic Acid Activation of the Alternative Oxidase of Plant Mitochondria.” FEBS Letters 329: 259–262. 8365467 10.1016/0014-5793(93)80233-k

[pbi70479-bib-0021] Miyashita, Y. , R. Dolferus , K. P. Ismond , and A. G. Good . 2007. “Alanine Aminotransferase Catalyses the Breakdown of Alanine After Hypoxia in *Arabidopsis thaliana* .” Plant Journal 49: 1108–1121. 10.1111/j.1365-313X.2006.03023.x 17319845

[pbi70479-bib-0022] Moczulski, D. , I. Majak , and D. Mamczur . 2009. “An Overview of Beta‐Oxidation Disorders.” Postepy Higieny I Medycyny Doswiadczalnej 63: 266–277. 19535822

[pbi70479-bib-0023] Porras‐Dominguez, J. , J. Lothier , A. M. Limami , and G. Tcherkez . 2024. “D‐Amino Acids Metabolism Reflects the Evolutionary Origin of Higher Plants and Their Adaptation to the Environment.” Plant, Cell & Environment 47, no. 5: 1503–1512. 10.1111/pce.14826 38251436

[pbi70479-bib-0024] Qiu, X. M. , Y. Y. Sun , X. Y. Ye , and Z. G. Li . 2019. “Signaling Role of Glutamate in Plants.” Frontiers in Plant Science 10: 1743. 32063909 10.3389/fpls.2019.01743 PMC6999156

[pbi70479-bib-0025] Selinski, J. , A. Hartmann , S. Höfler , G. Deckers‐Hebestreit , and R. Scheibe . 2016. “Refined Method to Study the Posttranslational Regulation of Alternative Oxidases From *Arabidopsis thaliana* In Vitro.” Physiologia Plantarum 157: 264–279. 26798996 10.1111/ppl.12418

[pbi70479-bib-0026] Seymour, R. S. , M. C. Barnhart , and G. A. Bartholomew . 1984. “Respiratory Gas Exchange During Thermogenesis In *philodendron selloum* Koch.” Planta 161: 229–232. 24253648 10.1007/BF00982917

[pbi70479-bib-0027] Seymour, R. S. , and P. G. D. Matthews . 2006. “The Role of Thermogenesis in the Pollination Biology of the Amazon Waterlily *Victoria amazonica* .” Annals of Botany 98: 1129–1135. 17018568 10.1093/aob/mcl201 PMC2803590

[pbi70479-bib-0028] Seymour, R. S. , and P. Schultze‐Motel . 1997. “Heat‐Producing Flowers.” Endeavour 21: 125–129.

[pbi70479-bib-0029] Sluse, F. E. , A. M. Almeida , W. Jarmuszkiewicz , and A. E. Vercesi . 1998. “Free Fatty Acids Regulate the Uncoupling Protein and Alternative Oxidase Activities in Plant Mitochondria.” FEBS Letters 433: 237–240. 9744802 10.1016/s0014-5793(98)00922-3

[pbi70479-bib-0030] Tang, S. , N. Guo , Q. Tang , et al. 2022. “Pyruvate Transporter BnaBASS2 Impacts Seed Oil Accumulation in *Brassica napus* .” Plant Biotechnology Journal 20: 2406–2417. 36056567 10.1111/pbi.13922 PMC9674310

[pbi70479-bib-0031] Tcherkez, G. , C. Abadie , C. Dourmap , J. Lalande , and A. M. Limami . 2024. “Leaf Day Respiration: More Than Just Catabolic CO_2_ Production in the Light.” Plant, Cell & Environment 47, no. 7: 2631–2639. 10.1111/pce.14904 38528759

[pbi70479-bib-0032] Van Aken, O. , E. Giraud , R. Clifton , and J. Whelan . 2009. “Alternative Oxidase: A Target and Regulator of Stress Responses.” Physiologia Plantarum 137: 354–361. 19470093 10.1111/j.1399-3054.2009.01240.x

[pbi70479-bib-0033] Vercesi, A. E. , J. Borecký , I. G. Maia , P. Arruda , I. M. Cuccovia , and H. Chaimovich . 2006. “Plant Uncoupling Mitochondrial Proteins.” Annual Review of Plant Biology 57: 383–404. 10.1146/annurev.arplant.57.032905.105335 16669767

[pbi70479-bib-0034] Walker, D. B. , J. Gysi , L. Sternberg , and M. J. Deniro . 1983. “Direct Respiration of Lipids During Heat Production in the Inflorescence of *Philodendron selloum* .” Science 220: 419–421. 17831415 10.1126/science.220.4595.419

[pbi70479-bib-0035] Wang, R. , L. Chen , Y. Jia , et al. 2022. “Heat Production and Volatile Biosynthesis Are Linked via Alternative Respiration in *Magnolia denudata* During Floral Thermogenesis.” Frontiers in Plant Science 13: 955665. 36311085 10.3389/fpls.2022.955665 PMC9614359

[pbi70479-bib-0036] Wang, R. , X. Liu , S. Mou , S. Xu , and Z. Zhang . 2013. “Temperature Regulation of Floral Buds and Floral Thermogenicity in *Magnolia denudata* (Magnoliaceae).” Trees 27: 1755–1762.

[pbi70479-bib-0037] Wang, R. , S. Xu , X. Liu , Y. Zhang , J. Wang , and Z. Zhang . 2014. “Thermogenesis, Flowering and the Association With Variation in Floral Odour Attractants in *Magnolia sprengeri* (Magnoliaceae).” PLoS One 9: e99356. 24922537 10.1371/journal.pone.0099356 PMC4055676

[pbi70479-bib-0038] Wang, S. , M. Yu , C. Liu , et al. 2025. “Integrating Mitoflash, Energy Substrate, and Hormone Analyses to Advance Understanding of Magnolia Endodormancy Mechanisms.” International Journal of Biological Macromolecules 315: 144333. 40389010 10.1016/j.ijbiomac.2025.144333

[pbi70479-bib-0039] Wang, S. , M. Yu , Z. Wang , et al. 2025. “Integrated Multiscale Imaging and Noninvasive Micro‐Sensing Decipher Spatiotemporal Calcium Dynamics in Thermogenic Tissue of Magnolia Flower.” *Plant, Cell and Environment*. 10.1111/pce.70196. 40988373

[pbi70479-bib-0040] Xiang, F. , W. C. Liu , X. Liu , et al. 2023. “Direct Balancing of Lipid Mobilization and Reactive Oxygen Species Production by the Epoxidation of Fatty Acid Catalyzed by a Cytochrome P450 Protein During Seed Germination.” New Phytologist 237: 2104–2117. 36495066 10.1111/nph.18669

[pbi70479-bib-0041] Xiao, R. , O. Youngjun , X. Zhang , N. N. Thi , H. Lu , and I. Hwang . 2023. “Osmotic Stress‐Induced Localisation Switch of CBR1 From Mitochondria to the Endoplasmic Reticulum Triggers ATP Production via β‐Oxidation to Respond to Osmotic Shock.” Plant, Cell & Environment 46: 3420–3432. 10.1111/pce.14671 37469026

[pbi70479-bib-0042] Xiao, W. , R. S. Wang , D. E. Handy , and J. Loscalzo . 2018. “NAD(H) and NADP(H) Redox Couples and Cellular Energy Metabolism.” Antioxidants & Redox Signaling 28: 251–272. 28648096 10.1089/ars.2017.7216 PMC5737637

[pbi70479-bib-0043] Yu, M. , S. Wang , G. Gu , et al. 2025. “Integration of Mitoflash and Time‐Series Transcriptomics Facilitates Energy Dynamics Tracking and Substrate Supply Analysis of Floral Thermogenesis in Lotus.” Plant, Cell & Environment 48: 893–906. 10.1111/pce.15185 39360569

[pbi70479-bib-0044] Yu, M. , S. Wang , L. Kong , et al. 2025. “A Combined Transcriptomic and Metabolomic Analysis Reveals the Metabolic Reprogramming of Developing Thermogenic Tissues in *Nelumbo nucifera* .” Plant Journal 122: e70193. 10.1111/tpj.70193 40327963

[pbi70479-bib-0045] Zayed, O. , O. A. Hewedy , A. Abdelmoteleb , et al. 2023. “Nitrogen Journey in Plants: From Uptake to Metabolism, Stress Response, and Microbe Interaction.” Biomolecules 13: 1443. 37892125 10.3390/biom13101443 PMC10605003

[pbi70479-bib-0046] Zhang, Y. , J. Zhu , M. Khan , et al. 2022. “Transcription Factors ABF4 and ABR1 Synergistically Regulate Amylase‐Mediated Starch Catabolism in Drought Tolerance.” Plant Physiology 191: 591–609. 10.1093/plphys/kiac428 PMC9806598 36102815

[pbi70479-bib-0047] Zhu, Y. , J. Lu , J. Wang , F. Chen , F. Leng , and H. Li . 2011. “Regulation of Thermogenesis in Plants: The Interaction of Alternative Oxidase and Plant Uncoupling Mitochondrial Protein.” Journal of Integrative Plant Biology 53: 7–13. 21205176 10.1111/j.1744-7909.2010.01004.x

