# Supplementary material for: Multi‐Omics Combined With Mitochondrial Feeding Assays Reveal a Novel Energy Metabolism Strategy for Floral Thermogenesis in Magnolia Driven by Synergistic Supply of Multiple Substrates
Source: Plant Biotechnol J. 2025 Nov 28;24(4):2169–83. doi: 10.1111/pbi.70479 (PMC13140744; doi:10.1111/pbi.70479)
Supplement: Supplementary file 1 — Figure S1: Development and thermogenesis dynamics of the M. denudata flowers. Figure S2:. Global analysis of lipid changes from S1 (the pre‐thermogenic stage) to S2 (the thermogenic peak stage) in M. denudata flowers. [file PBI-24-2169-s001.docx]

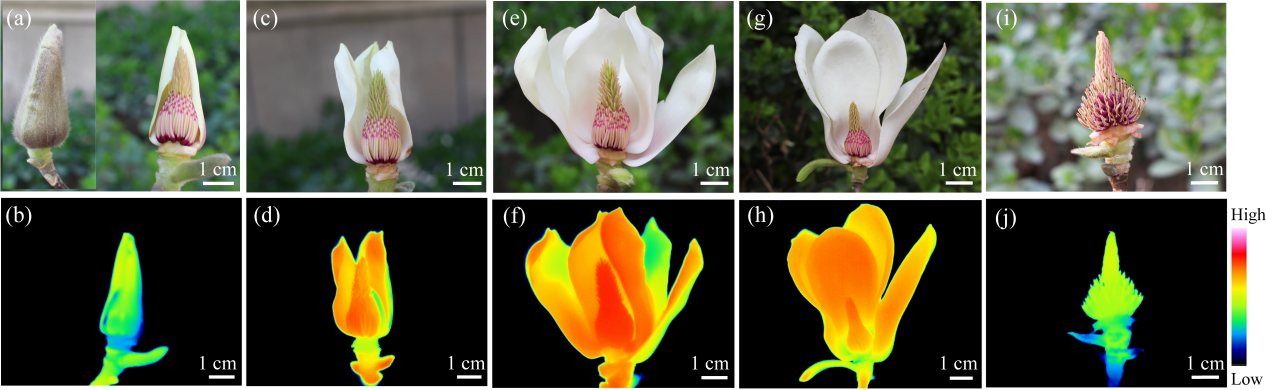


**Figure S1** Development and thermogenesis dynamics of the *M. denudata* flowers. (a, b) Stage 0: the petal unexposed stage. (c, d) Stage 1: the pre-thermogenic stage. (e, f) Stage 2: the thermogenic peak stage. (g, h) Stage 3: the post-thermogenic peak stage. (i, j) Stage 4: the wilting stage.


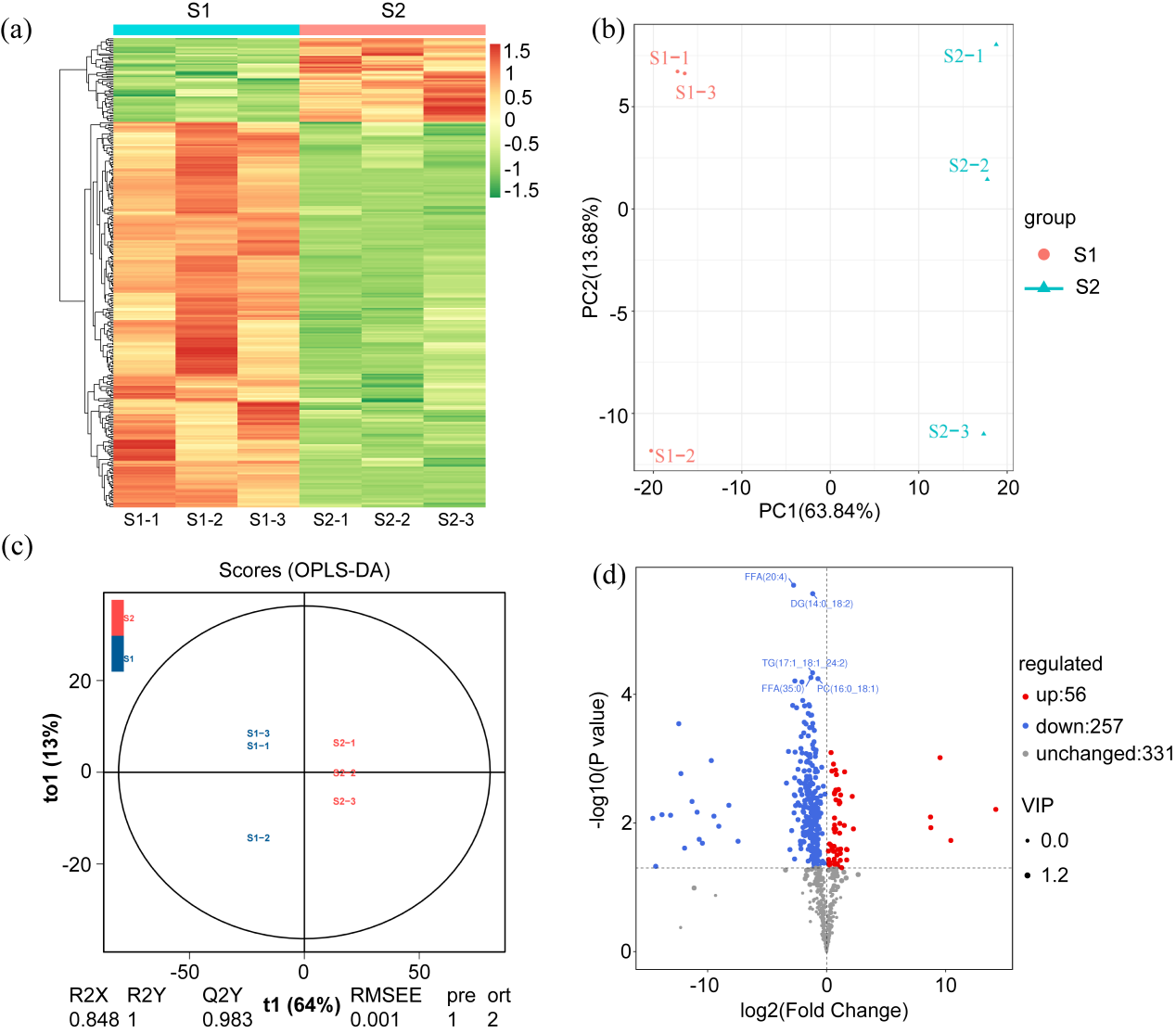


**Figure S2** Global analysis of lipid changes from S1 (the pre-thermogenic stage) to S2 (the thermogenic peak stage) in *M. denudata* flowers. Hierarchical cluster analysis (a), PCA analysis (b) and OPLS−DA analysis (c) of the total lipid class species in each *M. denudata* flower samples. (d) Volcano plot showing changes in lipid species from S1 to S2.
